# Pomegranate extract inhibits the interleukin-1β-induced activation of MKK-3, p38α-MAPK and transcription factor RUNX-2 in human osteoarthritis chondrocytes

**DOI:** 10.1186/ar3166

**Published:** 2010-10-18

**Authors:** Zafar Rasheed, Nahid Akhtar, Tariq M Haqqi

**Affiliations:** 1 Department of Medicine, Division of Rheumatology, MetroHealth Medical Center/Case Western Reserve University, 2500 Metro Health Drive, Cleveland, OH 44109, USA

## Abstract

**Introduction:**

Pomegranate has been revered throughout history for its medicinal properties. p38-MAPK is a major signal-transducing pathway in osteoarthritis (OA) and its activation by interleukin-1β (IL-1β) plays a critical role in the expression and production of several mediators of cartilage catabolism in OA. In this study we determined the effect of a standardized pomegranate extract (PE) on the IL-1β-induced activation of MKK3/6, p38-MAPK isoforms and the activation of transcription factor RUNX-2 in primary human OA chondrocytes.

**Methods:**

Human chondrocytes were derived from OA cartilage by enzymatic digestion, treated with PE and then stimulated with IL-1β. Gene expression of p38-MAPK isoforms was measured by RT-PCR. Western immunoblotting was used to analyze the activation of MAPKs. Immunoprecipitation was used to determine the activation of p38-MAPK isoforms. DNA binding activity of RUNX-2 was determined using a highly sensitive and specific ELISA. Pharmacological studies to elucidate the involved pathways were executed using transfection with siRNAs.

**Results:**

Human OA chondrocytes expressed p38-MAPK isoforms p38α, -γ and -δ, but not p38β. IL-1β enhances the phosphorylation of the p38α-MAPK and p38γ-MAPK isoforms but not of p38δ-MAPK isoform in human OA chondrocytes. Activation of p38-MAPK in human OA chondrocytes was preferentially mediated via activation of MKK3. In addition, we also demonstrate that polyphenol rich PE inhibited the IL-1β-induced activation of MKK3, p38α-MAPK isoform and DNA binding activity of the transcription factor RUNX-2.

**Conclusions:**

Our results provide an important insight into the molecular basis of the reported cartilage protective and arthritis inhibitory effects of pomegranate extract. These novel pharmacological actions of PE on IL-1β stimulated human OA chondrocytes impart a new suggestion that PE or PE-derived compounds may be developed as MKK and p38-MAPK inhibitors for the treatment of OA and other degenerative/inflammatory diseases.

## Introduction

Osteoarthritis (OA), the most common forms of arthritis, is a progressive degenerative joint disease that has a major impact on joint function and the patient's quality of life. This disorder is regulated by proinflammatory cytokines such as IL-1 and TNF-α that can activate a broad array of intracellular signal transduction mechanisms [1]. Of the cytokine- activated pathways, MAP kinases are especially important because they regulate the production of several mediators of inflammation and cartilage damage [2]. Members of the MAP kinase family phosphorylate a number of transcription factors including runt-related transcription factor-2 (RUNX-2), with subsequent activation of matrix metalloproteinases (MMPs) and inflammatory cytokine gene expressions [3]. p38-MAPK, in particular, can regulate cytokine production through a variety of transcriptional and translational mechanisms [4]. Furthermore, p38-MAPK participates in other inflammation-related events, such as neutrophil activation [5], apoptosis [6] and nitric oxide synthase induction [7]. Inhibition of p38-MAPK with the commonly used pharmacological agent SB203580 reduces proinflammatory cytokine production in monocytes/macrophages, neutrophils, and T lymphocytes [8]. In a rodent model of inflammatory arthritis, p38-MAPK inhibition suppressed the inflammation and bone destruction [9]. TNF-α for instance, activates preferentially the MAPK-p38 isoform p38α in synovial macrophages and synovial fibroblasts (RASFs) in rheumatoid arthritis (RA) [10]. In addition, p38α-MAPK is activated in human osteoclast precursor cells upon binding of RANKL, which indicates a possible role for p38α-MAPK isoform in bone destruction. Inhibition of p38-MAPK with small-molecule compounds has been successful for the treatment of experimental arthritis in different animal models [11,12]. Of note, inhibition of the p38α-MAPK isoform seems to be particularly effective with regard to cartilage and bone destruction, since a selective p38α-MAPK inhibitor reduced bone loss, numbers of osteoclasts and serum levels of cartilage breakdown metabolites in mice with CIA [12]. The upstream kinases, MKK3 and MKK6 are important regulators of p38-MAPK and represent potential therapeutic targets to modulate cytokine production [13]. Studies in MKK3 or MKK6 knockout mice demonstrate that both are essential for full p38-MAPK activation *in vivo *[14]. MKK3 selectively phosphorylates p38α-, γ-, and δ-MAPKs whereas MKK6 activates all four p38-MAPK isoforms (α, β, γ, and δ) [15]. This suggests that substrate selectivity might contribute to the distinct functional profiles of MKK activation. It is well documented that activation of the runt-related transcription factor RUNX-2 is mediated by activated p38-MAPK as inhibition of p38-MAPK abrogates its activity and the expression of cartilage degrading enzymes in chondrocytes [16]. These and other studies [17] clearly show that inhibition of specific MAPKs or transcription factor may be an effective approach for the inhibition of joint destruction in arthritis.

Pomegranate fruit (*Punica granatum L*) is revered through the ages for its medicinal properties. Pomegranate fruit extract (PFE) is a rich source of highly potent antioxidants and is widely used in several traditional medicinal systems for the treatment of inflammation and pain in arthritis and other diseases. Consistent with this notion, our published study is noteworthy where we showed for the first time that a standardized PFE preparation was non-toxic to human OA chondrocytes and prevented the IL-1β-induced cartilage breakdown by inhibiting the IL-1β-induced production of MMPs by blocking the activation of p38-MAPK and the transcription factor NF-κB in human OA chondrocytes [18]. In other studies, we have shown that bioavailable metabolites of PFE inhibited COX-2 activity in human OA chondrocytes [19], and that consumption of PFE suppressed inflammation and joint destruction in an animal model of inflammatory arthritis [20].

Therefore, based on our published data and the studies showing that IL-1β activates the human chondrocytes and induces the expression of mediators that play a major role in cartilage degradation in OA, we determined whether PE inhibits the IL-1β-induced cartilage degradation by modulating the activation of relevant signal transduction pathways and transcription factors in human OA chondrocytes. Our results show that a standardized pomegranate extract (PE) selectively inhibited the IL-1β-induced activation of p38α-MAPK isoform and its upstream regulator MKK3 in primary human OA chondrocytes. In addition, IL-1β-induced DNA binding activity of RUNX-2 was also inhibited by PE in primary human OA chondrocytes and correlated with the inhibition of p38α-MAPK isoform.

## Materials and methods

### Specimen selection and articular chondrocytes preparation

With Institutional Review Board (IRB) approval, discarded human cartilage samples were obtained from the knee joints of 19 OA patients aged 58 to 77 years (mean age, 62 ± 7.7 years; 14 female, 5 male Caucasians) who underwent joint replacement surgery at MetroHealth Medical Center, Cleveland, Ohio, USA. The macroscopic cartilage degeneration was determined by staining of femoral head samples with India ink [21] and the cartilage with smooth articular surface ("unaffected cartilage") was resected and used to prepare chondrocytes by enzymatic digestion as previously described [22-27]. Histological analysis of some of the "unaffected cartilage" samples was performed on 5 μM thick sections stained with H&E and Safranin O and graded using Mankin score [28]. Grading of the histology slides (not shown) revealed that all of the cartilage pieces taken from the unaffected area had a Mankin score of <3 for structure and Mankin score of 2 for cellularity. Isolated chondrocytes were plated at a density of 1 × 10^6^/ml in 35-mm tissue culture dishes (Corning, Corning, NY, USA) in complete DMEM medium as previously described [22].

### Chemical composition of PE

PE is produced in a two-step process: 1) extraction of fruit residue after pressing for juice and 2) solid-phase extraction to produce a powder with a high concentration of polyphenols. Extraction is performed during fruit harvest using pressed pomegranate fruit and arils. Composition of the powder extract (PE) used in this study has been described previously [18-20].

### Treatment of chondrocytes with IL-1β and PE

Human OA chondrocytes (1 × 10^6^/ml) were plated in 35 mm culture dishes (Becton-Dickinson, Franklin Lakes, NJ, USA) in complete DMEM with 10% fetal calf serum and allowed to grow for 72 h at 37°C and 5% CO_2 _in a tissue culture incubator. PE was dissolved in water and filter sterilized. Primary OA chondrocytes (>80% confluent) were serum starved for 12 hours/overnight and then were pre-treated with different doses of PE (6.25 to 100 μg/ml) for one or two hour and then stimulated with human recombinant IL-1β (# 201LB, R&D Systems, St Paul, MN, USA, 10 ng/ml) for 5 to 120 minutes. OA chondrocytes cultured without IL-1β or PE served as controls. Cell viability was determined using Trypan Blue and the cells were counted using a hemocytometer. All experiments were performed within four days of the initiation of primary culture to avoid dedifferentiation of human OA chondrocytes.

### Immunoprecipitation and Western immunoblotting

Stimulated and control human OA chondrocytes were washed with cold PBS and lysed using the cell lysis RIPA buffer (50 mM Tris:HCl, pH 7.5; 150 mM NaCl; 1% IGEPAL, 4 mM EDTA, 0.1% sodium deoxycholate; 10 mM Na_4_P_2_O_7_, 10 mM NaF, 2 mM Na_3_VO_4_, 1 mM PMSF, 1 μg/ml leupeptin, 1 μg/ml aprotinin). Cytoplasmic and nuclear fractions were prepared as previously described [29]. Anti-MKK-3 antibody (# 32-6900), anti-MKK-6 (#32-7100) and anti-p38β antibody (#33-8700) were purchased from Zymed (San Francisco, CA, USA); anti-phospho-MKK3/6 antibody (sc-7994-R) was from Santa Cruz Biotechnology (Santa Cruz, CA, USA) and the anti-RUNX2 antibody (#D130-3 ) was from MBL, Naka-ku Nagoya, Japan. Anti-p38γ antibody (#MAB1347) was from R&D Systems (Minneapolis, MN, USA); anti-phospho-p38MAPK antibody (#9215S), anti-p38α antibody (#9218), and anti-p38δ antibody (#9214) were from Cell Signaling Technologies (Amherst, Beverly, MA, USA). Proteins of interest were immunoprecipitated using 200 μg total cell lysate protein following standard protocols (Cell Signaling Technology). Briefly, cell lysate was pre-cleared with protein- A/G agarose (Pierce, Rockford, IL, USA) at 4°C and then incubated with the appropriate antibody overnight at 4°C, mixed with Protein A/G agarose, centrifuged and the complexes were washed with cold RIPA buffer prior to SDS-PAGE. Total cell lysate or nuclear/cytoplasmic fraction protein (35 μg/lane) or immunoprecipitated proteins were resolved by SDS-PAGE (10% resolving gel with 4% stacking) and transferred to PVDF immobilon-P transfer membranes (Cat. # IPVH00010, Millipore Corporation, Bedford, MA, USA). Membranes were blocked with non-fat dry milk powder in Tris buffered saline and 0.1% Tween-20 (TBS-T). Blots were probed with 1:200 to 1:1000 diluted primary antibodies specific for the target protein. Immunoreactive proteins were visualized by using 1:1000 diluted HRP-linked secondary antibodies and enhanced chemiluminescence (GE Healthcare, Milwaukee, WI, USA) [23]. Images were captured using AFP-Imaging System (Minimedical Series, Elms Ford, NY, USA).

### Reverse transcription PCR (RT-PCR)

To analyze the gene expression of the p38-MAPK isoforms α, β, γ and δ, total cellular RNA was prepared using a commercially available kit (Qiagen, Valencia, CA, USA). First-strand cDNA was synthesized using 1 μg total RNA and the SuperScript First Strand cDNA synthesis kit (Invitrogen, Carlsbad, CA, USA). Primers used for PCR assisted amplification were: p38α- MAPK (NM_139012, F 5'-GATCAGTTGAAGCTCATTTTAA-3'; R 5'-CACTTGAATAATATTTGGAGAGT-3', expected size of the DNA fragment 479 bp), p38β- MAPK (NM_002751, F 5'-AGCCATATCTGG CAA GAA GCT GGA-3'; R 5'-AAG TGT CCG AGT CCA AGT CCA CAT-3', expected size of the DNA fragment 262 bp); p38γ- MAPK (NM_002969, F 5'-TTG AAT TGG ATG CGC TAC ACG CAGB-3'; R 5'-AGG GCT TGC ATT GGT CAG GAT AGA-3', expected size of the DNA fragment 255 bp) and p38δ- MAPK (NM_002754, F 5'-TGT GCA GAA GCT GAA CGA CAA AGC-3'; R 5'-TGC CAT GCA AGA TGA GTC CCT ACA-3', expected size of the DNA fragment 380 bp). PCR reaction was carried out using the PTC-100 Thermal Cycler as follows: two minutes at 95°C, followed by 30 cycles of one minutes at 95°C, 30 seconds at 62°C, and one minute at 72°C. The amplified products were electrophoresed on 1.5% agarose gels in TAE buffer and visualized by ethidium bromide staining. Specificity of amplified fragments was verified by cloning and sequencing.

### KK3 or MKK6 knockdown by transfection with small interfering RNAs (siRNA)

Human OA chondrocytes were transfected with human MKK3 or MKK6-specific siRNAs using On-Target SMART Pools (# L-00350900, L-00396700, Dharmacon RNA Technologies, Lafayette, CO, USA). Human GAPDH control On-target SMART pool siRNA (# D-0018301020, Dharmacon) was used as negative control. Chondrocytes were transfected using Amaxa Human Chondrocytes Nucleofector Kit (# VPF-1001, Lonza, Walkersville, MD, USA) according to the manufacturer's instructions. In brief, human OA chondrocytes 1.4 × 10^6 ^were transfected with 30 nM-300 nM siRNA and with 100 μl nucleofector solution using electroporation program U-024 and the transfected OA chondrocytes were seeded in six-well plates. After 24 hours, the culture medium was changed to serum-free medium for the experiments using IL-1β. Transfection efficiency was monitored with red fluorescent siRNA oligonucleotides (siGLO red indicator, # D0016300220, Dharmacon). Approximately 70 to 80% of the chondrocytes emitted red fluorescence signal when transfected with siGLO.

### RUNX-2 DNA binding activity assay

Activation and DNA binding activity of RUNX-2 in human OA chondrocytes pretreated with PE (2 h) and then stimulated with IL-1β (30 minutes) was determined using a highly sensitive and specific Transcription Factor ELISA Kit according to the instructions of the manufacturer (#44496/44996, Active Motif North America, Carlsbad, CA, USA). Color intensity was read at 450 nm using Synergy HT ELISA plate reader (Biotek Instruments, Winooski, VT, USA).

### Treatment of human T cells HSB.2 with IL-1β, PHA and PE

To determine whether the inhibition of IL-1β-induced effects in human OA chondrocytes by PE was not due to an interference by PE in the receptor binding of exogenously added IL-1β, we performed a bioassay using the IL-1-dependent human T cell line HSB.2 known to produce high levels of IL-2 in an IL-1-dependent manner [30]. These cells were obtained from ATCC (CCL 120.1) and were cultured essentially as described [30]. Cells were stimulated with IL-1β, PHA (phytohemagglutinin) and PE and the production of IL-2 was quantified using the IL-2-specific ELISA according to the instructions of the manufacturer (R&D Systems). Plates were read at 450 nm using Synergy HT microplate reader (Biotek Instruments).

### Densitometric analysis

Measurements of the scanned bands were performed using UN-SCAN-IT software (Silk Scientific Corporation, Orem, UT, USA). Each band was scanned five times, and the mean band intensity (pixels per band) was obtained. Data were normalized to suitable loading controls and expressed as Mean ± SD followed by appropriate statistical analysis.

### Statistical analysis

All measurements were performed in duplicates and repeated at least three times using different age- and sex-matched OA samples. All statistical analyses were performed using Origin 6.1 software package (Northampton, MA, USA) (one paired two tailed *t*-test with one way ANOVA and Tukey's post-hoc analysis) and *P *< 0.05 was considered significant. Values shown are mean ± SD unless stated otherwise.

## Results

### PE was non-toxic to human OA chondrocytes *in vitro*

In these studies pre-treatment of human OA chondrocytes for up to 24 hr with varying concentrations of PE (up to 200 μg/ml) was found to be non-toxic and showed no effect on the cell viability (data not shown) as reported by us previously (18). Based on this data the maximum concentration of PE used in these studies was 100 μg/ml.

### Expression and activation of MKK3 and MKK6 in human OA chondrocytes

**Figure 1:**
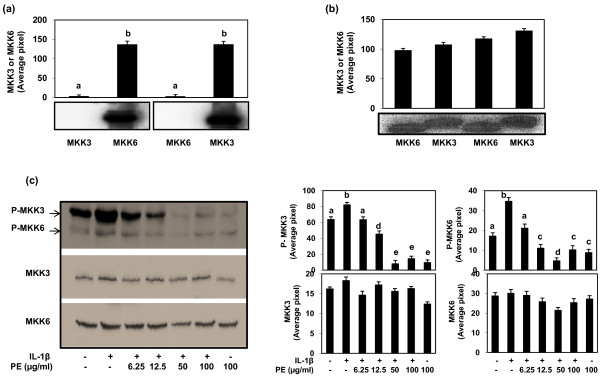
**Differential inhibition of IL-1β-induced activation of MKK3 and MKK6 by PE in human OA chondrocytes**. **(a) **Specificity of anti-MKK3 and anti-MKK6 monoclonal antibodies used in these studies was determined by using *in vitro *translated MKK3 and MKK6 proteins. No cross reactivity with the non-target protein was observed. **(b) **Expression of MKK3 and MKK6 in human chondrocytes. Human OA chondrocytes were grown to 70 to 80% confluence, serum starved overnight and then stimulated with IL-1β (5 ng/ml) for 15 minutes while control cultures received only the fresh medium. Unstimulated and stimulated human OA chondrocytes were lyzed and lysates were used for the immunoprecipitation of MKK3 and MKK6 using monoclonal antibodies. Immunoprecipitated protein was resolved on a 4 to 20% gradient gel by SDS-PAGE and Western blots were probed with anti-phosphoMKK3/6 antibodies. **(c) **Effect of PE on MKK3 and MKK6 phosphorylation in IL-1β-stimulated human OA chondrocytes. After treatment with PE (6.25 to 100 μg/ml) for 2 h at 37°C, primary human OA chondrocytes (70 to 80% confluent) were incubated with IL-1β (10 ng/ml) for 30 minutes, and then the phosphorylation of MKK3 and MKK6 was determined by immunoblotting using anti-phospho-MKK3 (Ser189)/anti-phospho-MKK6 (Ser207) antibody. Band images were digitally captured and the band intensities (pixels/band) were obtained using the Un-Scan-It software and are expressed as average pixels/band. The data shown are cumulative of two experiments. Average pixel values are presented as Mean ± SD; data without a common letter differ, *P *< 0.05.

We first determined the specificity of antibodies used for the detection and immunoprecipitation of MKK3 and MKK6 protein in human OA chondrocytes stimulated with IL-1β. We used the *in vitro *translated (TnT System, Promega, Madison, WI, USA) human MKK3 and MKK6 proteins (MKK3 pcDNA3FLAG and MKK6 pcDNA3 FLAG plasmids courtesy of Dr. Roger Davis) to determine the specificity of antibodies used. An aliquot of the translation mixture was resolved by SDS-PAGE and the blot was probed with either anti-MKK3 antibody or anti-MKK6 antibody (Figure 1a). These results show that the antibodies used in this study were highly specific for MKK3 and MKK6 protein as no cross reactivity with non-target protein was observed. We then used these antibodies to determine the expression and phosphorylation of MKK3 and MKK6 proteins in human OA chondrocytes. IL-1β stimulated and non-stimulated OA chondrocytes lysates were used to immunoprecipate (IP) MKK3 and MKK6 kinases using the specific antibodies and the IP proteins were resolved by SDS-PAGE and transferred to nitrocellulose membranes and the blots were probed with polyclonal anti-phospho-MKK3/MKK6 antibodies. Our results show that both MKK3 and MKK6 were constitutively phosphorylated in the primary human OA chondrocytes but the intensity of MKK3 phosphorylation was significantly enhanced (*P *< 0.05) after stimulation with IL-1β (Figure 1b &1c). These results thus indicate that IL-1β preferentially activates MKK3 in human OA chondrocytes.

### Effect of PE on IL-1β-induced activation of MKK3 and MKK6 in human OA chondrocytes

To determine the effect of PE on IL-1β-induced activation of MKK3 and MKK6 in human OA chondrocytes, cells were pretreated with PE (6.25 to 100 μg/ml) for two hours and then stimulated with IL-1β for 30 minutes and cell lysate was analyzed by Western immunoblotting. Treatment of primary human OA chondrocytes with PE attenuated the IL-1β-induced phosphorylation of both MKK3 and MKK6 in a dose dependent manner with the maximum inhibitory effect of PE being at 50 μg/ml concentration (Figure 1c). Higher concentrations produced no additional suppression of phosphorylation of either MKK3 or MKK6 in human OA chondrocytes.

### Effect of MKK3 or MKK6 knock down on IL-1β-induced activation of p38-MAPK in human OA chondrocytes

**Figure 2:**
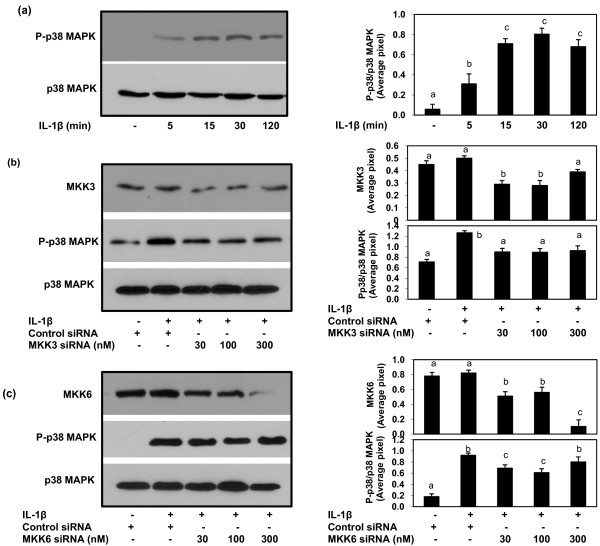
**Activation profile of p38-MAPK in IL-1β-stimulated primary human OA chondrocytes**. **(a) **Primary human OA chondrocytes (70 to 80% confluent) were incubated with IL-1β (10 ng/ml) for 5 to 120 minutes, and then the phosphorylation of p38-MAPK was determined by Western immunoblot analysis using primary antibodies specific for phospho-p38-MAPK and p38-MAPK. **(b) **SiRNA-mediated knockdown of MKK3 and p38-MAPK phosphorylation in IL-1β-stimulated human OA chondrocytes. MKK3-siRNA (30 to 300 nM) transfected human OA chondrocytes were incubated with IL-1β (10 ng/ml) for 30 minutes, then the phosphorylation of p38-MAPK was determined by Western immunoblot analysis. **(c) **SiRNA-mediated knockdown of MKK6- and p38-MAPK phosphorylation in IL-1β-stimulated human OA chondrocytes. MKK6-siRNA (30 to 300 nM) transfected human OA chondrocytes were incubated with IL-1β (10 ng/ml) for 30 minutes, then the phosphorylation of p38-MAPK was determined by Western immunoblotting. Band images were digitally captured and the band intensities were obtained using UN-San-It software and are expressed in average pixels/band. Data shown are cumulative of two experiments. Average pixel values presented as Mean ± SD; data without a common letter differ, *P *< 0.05.

We first examined the activation profile of p38-MAPK in primary human OA chondrocytes in response to IL-1β. Human OA chondrocytes treated with IL-1β (10 ng/ml) for different time periods (5 to 120 minutes) phosphorylated the p38-MAPK protein (Figure 2a). Next, we investigated the effect of MKK3 or MKK6 knock down on the phosphorylation of p38-MAPK in response to stimulation with IL-1β in human OA chondrocytes. Transfection of human OA chondrocytes with MKK3 or MKK6-specific siRNAs knocked down the expression of target proteins and inhibited the IL-1β-induced activation of p38-MAPK (Figure 2b, c), but the inhibition of phosphorylation was more pronounced when the expression of MKK3 was knocked down compared to the knockdown of MKK6 (Figure 2b).

### Expression of p38-MAPK isoforms in human OA chondrocytes

**Figure 3:**
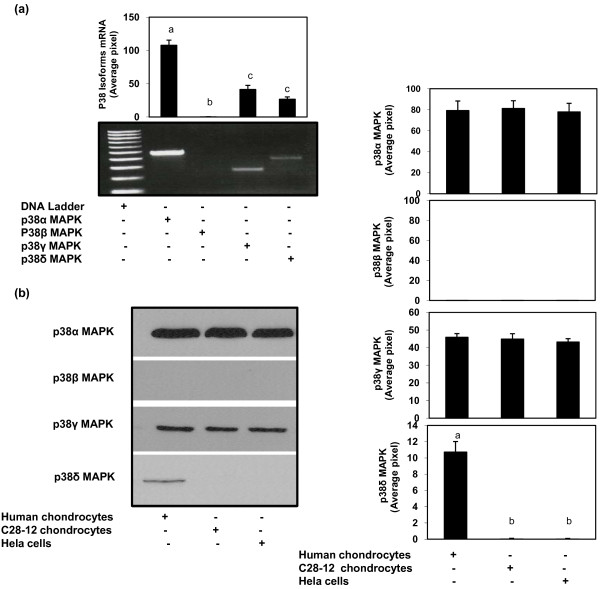
**Gene expression of p38-MAPK isoforms in primary human OA chondrocytes**. **(a)** Total RNA was prepared from the human OA chondrocytes, single stranded cDNA was synthesized and the PCR reaction was carried out using specific primers for p38-MAPKα, β, γ and δ isoforms as described in methods section above. **(b) **Protein expression of p38-MAPK isoforms in human OA chondrocytes, C28-I2 human chondrocytes and Hela cells. Western blot analysis was performed as described under Figure 1. Band images were digitally captured and the band intensities (pixels/band) were obtained using the Un-Scan-It software. Data shown are cumulative of two experiments. Average pixel values presented as Mean ± SD; data without a common letter differ, *P *< 0.05.

We next examined the gene expression pattern of p38-MAPK isoforms in primary human OA chondrocytes by PCR using the gene specific primers (see method section). Our results show that expression of p38α, p38γ and p38δ mRNA was clearly detected but the mRNA expression of p38β was not detected in primary human OA chondrocytes (Figure 3a). We then analyzed the protein expression of p38α, p38β, p38γ and p38δ isoforms in human OA chondrocytes, in Hela cells and in the chondrocytic cell line C28-I2 using commercially available antibodies (Cell Signaling; R&D Systems). Specificity of antibody binding was determined by using commercially available recombinant p38α, p38β, p38γ and p38δ proteins (Upstate/Millipore, Billerica, MA, USA) and by competition with inhibitory peptides (results not shown). Ours results show that in human OA chondrocytes expression of p38α, p38γ and p38δ proteins was readily detected but the expression of p38β protein was not detected either in the human OA chondrocytes or the chondrocytic cell line C28-I2 or in Hela cells (Figure 3b). Interestingly, the expression of p38β protein was undetectable in all the three tested cell types. Ability and specificity of anti-p38β antibody to detect the p38β protein was verified by using the same antibody to probe a Western blot prepared with the cell lysates of PC3 cells, MCF-7 cells and DU cells and a clear band of approximately 38 KD was detectible on the immunoblots (data not shown). We thus conclude that human OA chondrocytes do not express the p38β-MAPK .

### Effect of PE and IL-1β-induced activation of p38-MAPK isoforms in human OA chondrocytes

**Figure 4:**
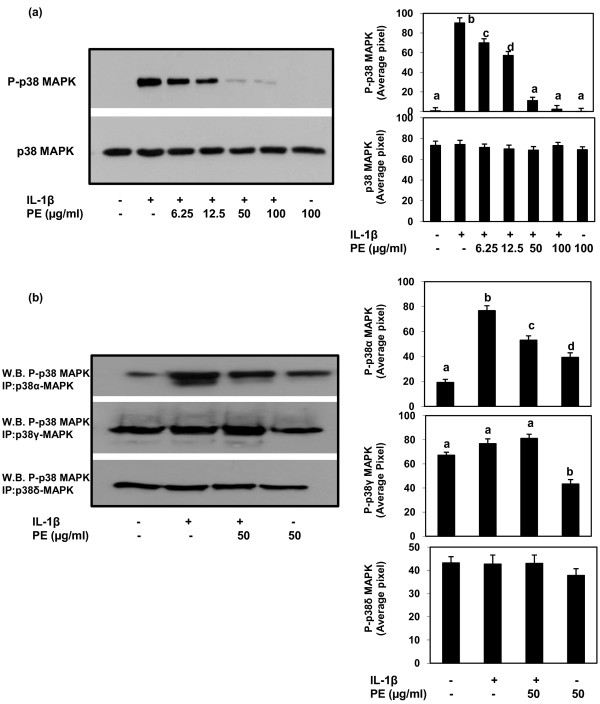
**Inhibition of IL-1β-stimulated phosphorylation of p38-MAPK by PE in primary human OA chondrocytes**. **(a) **Primary human OA chondrocytes were pretreated with PE for 2 h and then stimulated with IL-1β for 30 minutes and total cell lysate proteins were resolved by SDS-PAGE and analyzed by Western immunoblotting using primary antibodies specific for phospho-p38-MAPK and total p38-MAPK. **(b) **Inhibition of IL-1β-stimulated phosphorylation of p38-MAPK isoforms by PE in primary human OA chondrocytes. Primary human OA chondrocytes were pretreated with PE for 2 h and then stimulated with IL-1β for 30 minutes and total cell lysate were used to immunoprecipitate p38-α, γ and δ -MAPKs using monoclonal antibodies specific for each isoform as described above. Immunoprecipited protein was resolved by SDS-PAGE and Western blots were probed with anti-phospho-p38-MAPKantibody. Band images were digitally captured and the band intensities (pixels/band) were obtained as described under Figure 1. Average pixel values presented as mean ± SD; data without a common letter differ, *P *< 0.05.

We next examined the effect of PE on IL-1β-induced activation of p38-MAPK isoforms in human OA chondrocytes. Primary human OA chondrocytes (70 to 80% confluent) were pretreated with PE and then stimulated with IL-1β for 30 minutes and the cell lysates were analyzed by Western immunoblotting using the phospho-p38-MAPK and the total p38-MAPK monoclonal antibodies (Cell Signaling Technology). Results showed that human OA chondrocytes treated with IL-1β alone had higher level of p38-MAPK phosphorylation compared with unstimulated OA chondrocytes. However, IL-1β-induced phosphorylation of p38-MAPK was inhibited in chondrocytes pre-treated with PE (Figure 4a). Next, we examined which p38-MAPK isoform was activated by IL-1β in human OA chondrocytes. PE pretreated and IL-1β stimulated human OA chondrocytes lysates were used to immunoprecepitate the p38α, γ and δ isoforms using the commercially available antibodies (Cell Signaling Technology; R&D Systems). Immunoprecipitated proteins were resolved on a 4 to 20% gradient gel by SDS-PAGE and the Western blots were probed with the anti-phospho-p38MAPK antibody (Cell Signaling Technology). Our results showed that IL-1β predominantly activated the p38α-MAPK isoform as determined by significantly higher intensity of phosphorylation compared to other expressed isoforms (Figure 4b, *P *< 0.0001). It is also important to note that the IL-1β-induced activation of p38α-MAPK was inhibited by PE (*P *< 0.05) in primary human OA chondrocytes (Figure 4b).

### Effect of PE on IL-1β-induced activation of transcription factor RUNX-2 in human OA chondrocytes

**Figure 5:**
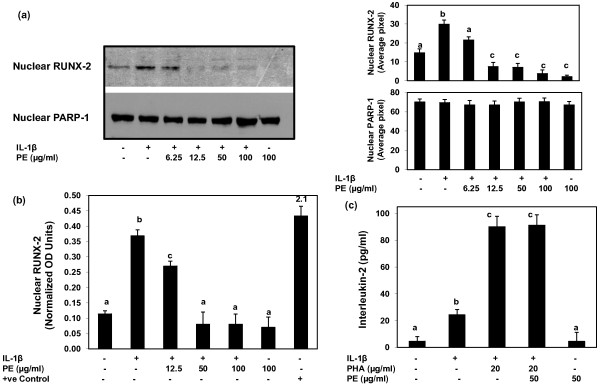
**PE inhibited the activation of RUNX-2 in primary human OA chondrocytes**. **(a) **Primary human OA chondrocytes were pretreated with PE for 2 h and stimulated with IL-1β for 30 minutes and the presence of activated RUNX-2 in the nucleus was determined by Immunoblotting. Protein loading was verified by using an antibody against the nuclear protein poly (ADP-ribose) polymerase-1 (PARP-1). Band images were digitally captured and the band intensities (pixels/band) were obtained as described under Figure 1. Average pixel values presented as mean ± SD. **(b) **PE inhibited the DNA binding activity of RUNX-2 in IL-1β-stimulated human OA chondrocytes. DNA binding activity of activated RUNX-2 present in the nucleus was determined by using a Transcription Factor ELISA kit specific for RUNX-2 (Active Motif). The Saos-2 nuclear extract supplied with the kit was used as a positive control. Data is representative of two experiments and is presented as Mean ± SEM. Values differ without a common letter *P *< 0.05. **(c) **PE does not block the interaction of IL-1β with IL-1 receptor. Human T cells HSB.2 (70 to 80% confluent) were stimulated with IL-1β and PHA in the presence or absence of PE and the production of IL-2 was quantified by a sandwich ELISA (R&D Systems) and the values were calculated from a standard curve. Data presented as Mean ± SEM; data without a common letter differ, *P *< 0.05.

Human OA chondrocytes were stimulated with IL-1β for 30 minutes as described above and the activation of RUNX-2 was analyzed by Western immunoblotting (Figure 5a). Analysis of the immunoblot revealed that the level of RUNX-2 was high in the nuclear extract from human OA chondrocytes treated with IL-1β (Figure-5a), when compared with the level detected in untreated human OA chondrocytes where RUNX-2 was barely detectable in the nucleus. Importantly, IL-1β stimulated increased RUNX-2 protein expression in the nucleus of human OA chondrocytes was inhibited by PE to less than the basal level (Figure 5a).

To determine whether PE also inhibits the IL-1β-induced DNA binding activity of the transcription factor RUNX-2 we used a sandwich ELISA assay kit. Exposure of OA chondrocytes to IL-1β significantly enhanced the DNA binding activity of RUNX-2 compared to controls (Figure 5b; *P *< 0.001). This IL-1β-induced increase in the DNA binding activity of RUNX-2 was inhibited significantly by PE in a dose-dependent manner (12.5 to 100 μg/ml) with the maximum inhibitory effect of PE being at 50 μg/ml concentration (Figure 5b). Higher concentrations produced no additional suppression of IL-1β-induced DNA binding activity of RUNX-2 in human OA chondrocytes.

### PE does not block the interaction of IL-1β with IL-1 Receptor

To determine whether the observed inhibition of IL-1β-induced activation of p38α-MAPK and RUNX-2 in human OA chondrocytes by PE was not due to an interference by endogenously added PE in the culture medium, we performed a bioassay using the IL-1-dependent human T cell line HSB.2 shown to produce high levels of IL-2 in an IL-1-dependent manner [30]. Human HSB.2 cells were stimulated with IL-1β + PHA and PE and the production of IL-2 was quantified by a sandwich ELISA (R&D Systems). Results presented in Figure 5c clearly demonstrate that PE did not interfere with the binding of IL-1β with IL-1R in these cells.

## Discussion

Progression of OA is characterized by destruction of the articular cartilage and other soft tissues in the joint. Tissue destruction is accompanied by remodeling and hypertrophy of neighboring bone and varying degrees of synovial inflammation [31]. The destruction, remodeling, and inflammation of joint tissues lead to joint failure manifested as joint pain and loss of function. Chondrocytes are the only cellular components of cartilage, and in pathologic conditions such as OA, chondrocytes increase the production of proteolytic enzymes such as aggrecanases and MMPs, resulting in aberrant cartilage destruction [32].

Intracellular signaling pathways provide the essential link between receptor signals and nuclear gene transcription. Key to these pathways are the evolutionarily conserved mitogen-activated protein kinases (MAPKs); of these, p38-MAPK (also known as MAPK14) has received considerable attention as potential therapeutic target for inflammatory and degenerative diseases such as OA [33]. p38-MAPK is thought to be crucial because selective p38-MAPK inhibitors block joint inflammation and destruction in several animal models of arthritis [12]. Four p38-MAPK isoforms (p38-α, -β, -γ, and -δ) have been characterized [34]. Although the relative expression of p38-MAPK isoforms in osteoarthritis has not been fully explored, but the best-studied is p38α-MAPK, which can be phosphorylated in many inflammatory cell lineages as well and regulates cytokine production by macrophages [34] and is known to be especially prevalent at sites of joint destruction [9].

**Figure 6:**
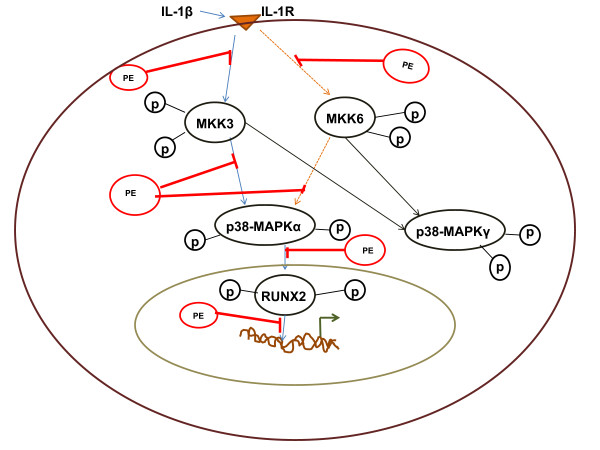
**PE inhibits IL-1β-induced signaling cascade in human OA chondrocytes**. The MAPKs are a group of signaling molecules that include the p38-MAPKs which are activated by upstream MAPK kinases MKK3 and MKK6. In human OA chondrocytes, IL-1β through IL-1R activates preferentially MKK3 which leads to the activation of p38-MAPK → RUNX2 pathway. Polyphenols rich PE inhibited the IL-1β-induced activation of MKK# and p38-MAPK in human OA chondrocytes. Breaking lines indicate reduced phosphorylation. IL-1R, IL-1β receptor; MAPK, mitogen-activated protein kinase; MKK, MAPK kinases; MMPs, matrix metalloproteinases; p, phosphorylation; PE, pomegranate extract; RUNX2, runt-related transcription factor-2.

In the present study we have shown for the very first time that human OA chondrocytes expressed mRNAs for p38-MAPK isoforms p38α, -γ, and -δ, but not of p38β isoform. This was further confirmed by analyzing the expression pattern of p38α, p38β, p38γ and p38δ isoforms at the protein level in human OA chondrocytes, Hela cells and in a chondrocytic cell line C28I2. C28I2 cells are transformed human chondrocytes, express a number of chondrocytes-specific markers and have been used previously [35]. Interestingly, in these studies also the expression of p38β-MAPK isoform was undetectable in human OA chondrocytes, HeLa cells and the C28-I2 chondrocytes indicating that these cell types do not express p38β isoform. In addition, we also determined the effect of IL-1β on the activation of p38-MAPK isoforms in primary human OA chondrocytes. Our results showed that IL-1β activates p38α-MAPK and p38γ-MAPK isoforms but not the p38δ-MAPK isoform in human OA chondrocytes. These are novel findings and have not been reported previously. Although, when over expressed in transfected cells, each p38 family member can be activated by IL-1β [36], however, activation of endogenously expressed p38-MAPK family members by IL-1β in human OA chondrocytes has not been reported. p38-MAPK was not activated by treatment with PE alone but pretreatment of human OA chondrocytes with PE selectively inhibited the IL-1β-induced activation of p38α-MAPK isoform. Furthermore, in the present study we also determined the potential role of MKK3 and MKK6 in IL-1β-induced activation of p38-MAPK in human OA chondrocytes. Both MKKs 3 and 6 are known to be phosphorylated in inflamed synovium and are constitutively expressed in fibroblast like synoviocytes (FLS) [37]. It is also documented in FLS, that MKK3 and MKK6 each form stable complexes with p38-MAPK that can activate transcriptional activities after cytokine exposure. For instance, MKK6 is the major activator of p38-MAPK in cells exposed to osmotic stress [13] and MKK3 is required for full activation of p38-MAPK in murine embryonic fibroblasts [38]. Expression of MKK3 and MKK6 and their relative contribution to the activation of p38-MAPK isoforms in OA has not been reported. Our results indicated that although both MKK3 and MKK6 were constitutively phosphorylated in the human OA chondrocytes (probably refecting the pre-activated state), but the effect of IL-1β was more pronounced on the phosphorylation of MKK3 as compared to MKK6 suggesting that MKK3 may be the primary kinase involved in the activation of p38-MAPK in human OA chondrocytes. This is supported by our results showing that siRNA-mediated knockdown of MKK3 significantly inhibited the IL-1β-induced activation of p38-MAPK compared to the level noted in cells with knockdown expression of MKK6. Next, we tested the ability of polyphenols rich PE on IL-1β-induced activation of MKK3 and MKK6 in primary human OA chondrocytes. Our results showed that PE inhibited the IL-1β-induced phosphorylation/activation of both MKK3 and MKK6, but the effect was more pronounced on MKK3 in primary human OA chondrocytes. Thus, treatment with PE affects particularly the activation of MKK3 which in turn has an impact on the activation of p38-MAPK downstream (summarized in Figure 6).

MAPKs and transcription factor NF-κB are important targets of polyphenols such as ellagic acid and quercetin found in PE [18,39]. Whereas the free radical scavenging and antioxidant properties of PE are well established [39], emerging literature reports suggest that their anti-arthritic effects may also be ascribed to their ability to modulate many signal transduction pathways including p38-MAPK and NF-κB in a manner that favors inhibition of OA and other inflammatory diseases [18-20,27]. It is well established that in chondrocytes activation of RUNX-2 is mediated by activated p38-MAPK [16]. IL-1 is a potent stimulator of many inflammatory genes in chondrocytes, and its effects are diminished in cells expressing a dominant-negative mutant of RUNX-2 [40]. Since PE suppressed the IL-1β-induced activation of p38-MAPK, we determined whether PE also inhibits IL-1β-induced activation of RUNX-2 in human OA chondrocytes. Pretreatment of human OA chondrocytes with PE inhibited the IL-1β- induced activation and DNA binding activity of RUNX-2 in a dose dependent manner. These data indicate that PE attenuates the inflammatory stimuli-induced activation and DNA binding activity of RUNX-2 in human OA chondrocytes.

In summary, this study shows that human OA chondrocytes expressed p38-MAPK isoforms p38α, γ and δ, but not p38β. Treatment of human OA chondrocytes with IL-1β activates p38α-MAPK and p38γ-MAPK but not p38δ-MAPK isoform. In addition, our results also showed that IL-1β activates the p38-MAPK preferentially via the upstream kinase MKK3 compared to MKK6 in human OA chondrocytes. In addition, we also investigated the effect of PE on IL-1β induced activation and DNA binding activity of the transcription factor RUNX2 in human OA chondrocytes. Our results demonstrate that PE suppresses the IL-1β-induced activation of RUNX-2 and this correlates with the inhibition of p38-MAPK activation. However, it is not known whether the inhibition of p38-MAPK - RUNX-2 pathway is related to the antioxidant activity of PE or one or more of the bioavailable constituents of PE exert this inhibitory effect by interfering with the kinase activity of p38-MAPK or both. This will be pursued in future studies.

## Conclusions

The present article is the first report demonstrating that IL-1β preferentially activates MKK3 → p38-MAPK → RUNX-2 pathway in human OA chondrocytes. This study also demonstrated that pretreatment of human OA chondrocytes with PE inhibited the IL-1β-induced activation of the upstream kinase MKK3 resulting in the inhibition of p38α-MAPK isoform and the activation and DNA binding activity of the transcription factor RUNX-2. Thus, our results suggest that PE or PE-derived compounds may be useful in blocking the activation of MKK3 and might provide the benefit of p38-MAPK inhibition and its downstream targets such as RUNX-2 without the direct inhibition of p38-MAPK in OA. This in turn may be of value in the development of MKK inhibitors for the treatment of OA and other degenerative diseases.

## Abbreviations

FLS: fibroblast like synoviocytes; IL-1β: interleukin-1β; IRB: Institutional Review Board; MAPK: mitogen activated protein kinase; MKK: MAPK kinases; MMPs: matrix metalloproteinases; OA: osteoarthritis; PE: pomegranate extract; PFE: pomegranate fruit extract; PHA: phytohemagglutinin; RA: rheumatoid arthritis; RASFs: synovial fibroblasts; RUNX-2: runt-related transcription factor-2

## Competing interests

The authors declare that they have no competing interests.

## Authors' contributions

ZR carried out the experimental work, collection, interpretation and manuscript drafting. NA carried out the experimental work. TMH conceived of the study, its design, coordinated, data interpretation and manuscript drafting. All authors have read and approved the final manuscript.
